# Supplementary material for: Zinc finger protein Zfp335 is required for the formation of the naïve T cell compartment
Source: eLife. 2014 Oct 24;3:e03549. doi: 10.7554/eLife.03549 (PMC4371841; doi:10.7554/eLife.03549)
Supplement: Supplementary file 1. — Hierarchical clustering analysis of 108 genes differentially expressed (p < 0.05, fold-change > 1.2) in blt/blt vs. WT mature CD4SP thymocytes and CD4+ RTEs. DOI: http://dx.doi.org/10.7554/eLife.03549.023 [file elife03549s001.pdf]

Supplementary File 1: Genes differentially expressed in mature CD4SP thymocytes and RTEs (*blt/blt* vs. WT)

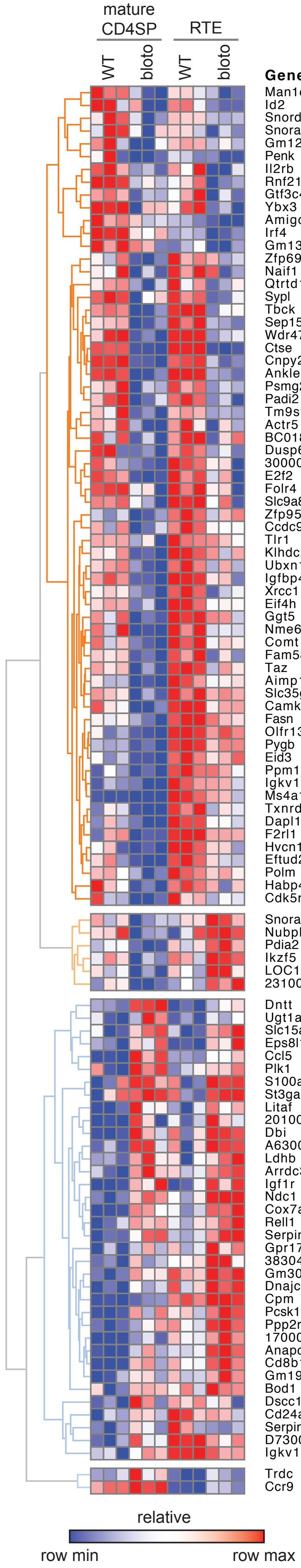

Gene ID	Gene description
Man1c1	mannosidase, alpha, class 1C, member 1
Id2	inhibitor of DNA binding 2
Snord52	small nucleolar RNA, C/D box 52
Snora20	small nucleolar RNA, H/ACA box 20
Gm129	predicted gene 129
Penk	preproenkephalin
Il2rb	interleukin 2 receptor, beta chain
Rnf213	ring finger protein 213
Gtf3c4	general transcription factor IIIC, polypeptide 4
Ybx3	Y box protein 3
Amigo2	adhesion molecule with Ig like domain 2
Irf4	interferon regulatory factor 4
Gm13139	predicted gene 13139
Zfp69	zinc finger protein 69
Naif1	nuclear apoptosis inducing factor 1
Qtrtd1	queuine tRNA-ribosyltransferase domain containing 1
Sypl	synaptophysin-like protein
Tbck	TBC1 domain containing kinase
Sep15	selenoprotein
Wdr47	WD repeat domain 47
Ctse	cathepsin E
Cnpy2	canopy 2 homolog (zebrafish)
Ankle2	ankyrin repeat and LEM domain containing 2
Psmg2	proteasome (prosome, macropain) assembly chaperone 2
Padi2	peptidyl arginine deiminase, type II
Tm9sf1	transmembrane 9 superfamily member 1
Actr5	ARP5 actin-related protein 5
BC018507	cDNA sequence BC018507
Dusp6	dual specificity phosphatase 6
3000002C10Rik	glyceraldehyde-3-phosphate dehydrogenase pseudogene
E2f2	E2F transcription factor 2
Folr4	folate receptor 4 (delta)
Slc9a8	solute carrier family 9 (sodium/hydrogen exchanger), member 8
Zfp954	zinc finger protein 954
Ccdc91	coiled-coil domain containing 91
Tlr1	toll-like receptor 1
Klhdc2	kelch domain containing 2
Ubxn11	UBX domain protein 11
Igfbp4	insulin-like growth factor binding protein 4
Xrcc1	X-ray repair complementing defective repair in Chinese hamster cells 1
Eif4h	eukaryotic translation initiation factor 4H
Ggt5	gamma-glutamyltransferase 5
Nme6	NME/NM23 nucleoside diphosphate kinase 6
Comt	catechol-O-methyltransferase
Fam58b	family with sequence similarity 58, member B
Taz	tafazzin
Aimp1	aminoacyl tRNA synthetase complex-interacting multifunctional protein 1
Slc35g1	solute carrier family 35, member G1
Camk2b	calcium/calmodulin-dependent protein kinase II, beta
Fasn	fatty acid synthase
Olfr1331	olfactory receptor 1331
Pygb	brain glycogen phosphorylase
Eid3	EP300 interacting inhibitor of differentiation 3
Ppm1j	protein phosphatase 1J
Igkv1-135	immunoglobulin kappa variable 1-135
Ms4a1	membrane-spanning 4-domains, subfamily A, member 1
Txnrd2	thioredoxin reductase 2
Dapl1	death associated protein-like 1
F2rl1	coagulation factor II (thrombin) receptor-like 1
Hvcn1	hydrogen voltage-gated channel 1
Eftud2	elongation factor Tu GTP binding domain containing 2
Polm	polymerase (DNA directed), mu
Habp4	hyaluronic acid binding protein 4
Cdk5rap1	CDK5 regulatory subunit associated protein 1
Snora34	small nucleolar RNA, H/ACA box 34
Nubpl	nucleotide binding protein-like
Pdia2	protein disulfide isomerase associated 2
Ikzf5	IKAROS family zinc finger 5
LOC100504779	uncharacterized LOC100504779
2310042E22Rik	RIKEN cDNA 2310042E22 gene
Dntt	deoxynucleotidyltransferase, terminal
Ugt1a9	UDP glucuronosyltransferase 1 family, polypeptide A9
Slc15a2	solute carrier family 15 (H+/peptide transporter), member 2
Eps8l1	EPS8-like 1
Ccl5	chemokine (C-C motif) ligand 5
Plk1	polo-like kinase 1
S100a10	S100 calcium binding protein A10 (calpactin)
St3gal6	ST3 beta-galactoside alpha-2,3-sialyltransferase 6
Litaf	LPS-induced TN factor
2010015L04Rik	RIKEN cDNA 2010015L04 gene
Dbi	diazepam binding inhibitor
A630033H20Rik	RIKEN cDNA A630033H20 gene
Ldhb	lactate dehydrogenase B
Arrdc3	arrestin domain containing 3
Igf1r	insulin-like growth factor I receptor
Ndc1	NDC1 transmembrane nucleoporin
Cox7a2l	cytochrome c oxidase subunit VIIa polypeptide 2-like
Rell1	RELT-like 1
Serpinb6b	serine (or cysteine) peptidase inhibitor, clade B, member 6b
Gpr174	G protein-coupled receptor 174
3830403N18Rik	RIKEN cDNA 3830403N18 gene
Gm3086	RuvB-like protein 1 pseudogene
Dnajc15	DnaJ (Hsp40) homolog, subfamily C, member 15
Cpm	carboxypeptidase M
Pcsk1	proprotein convertase subtilisin/kexin type 1
Ppp2r3a	protein phosphatase 2, regulatory subunit B, alpha
1700030C12Rik	RIKEN cDNA 1700030C12 gene
Anapc7	anaphase promoting complex subunit 7
Cd8b1	CD8 antigen, beta chain 1
Gm19503	cytochrome c oxidase subunit 7B, mitochondrial-like
Bod1	biorientation of chromosomes in cell division 1
Dscc1	defective in sister chromatid cohesion 1 homolog (S. cerevisiae)
Cd24a	CD24a antigen
Serpina1c	serine (or cysteine) peptidase inhibitor, clade A, member 1C
D730003I15Rik	RIKEN cDNA D730003I15 gene
Igkv10-94	immunoglobulin kappa variable 10-94
Trdc	T cell receptor delta, constant region
Ccr9	chemokine (C-C motif) receptor 9
